# A Constraint-Based Model Analysis of Enterocyte Mitochondrial Adaptation to Dietary Interventions of Lipid Type and Lipid Load

**DOI:** 10.3389/fphys.2018.00749

**Published:** 2018-06-15

**Authors:** Neeraj Sinha, Maria Suarez-Diez, Guido J. E. J. Hooiveld, Jaap Keijer, Vitor Martin dos Santos, Evert M. van Schothorst

**Affiliations:** ^1^Nutrition, Metabolism and Genomics, Division of Human Nutrition, Wageningen University & Research, Wageningen, Netherlands; ^2^Laboratory of Systems and Synthetic Biology, Wageningen University & Research, Wageningen, Netherlands; ^3^Human and Animal Physiology, Wageningen University & Research, Wageningen, Netherlands; ^4^LifeGlimmer GmbH, Berlin, Germany

**Keywords:** enterocytes, mitochondria, constraint-based metabolic model, mitochondrial dynamics, omega-3 lipids, high fat diet

## Abstract

Computational modeling of mitochondrial adaptability and flexibility in the small intestine upon different nutritional exposures will provide insights that will help to define healthy diet interventions. Therefore, a murine enterocyte-specific mitochondrial constraint-based metabolic model (named *MT_mmuENT127*) was constructed and used to simulate mitochondrial behavior under different dietary conditions, representing various levels and composition of nutrients absorbed by the enterocytes in mice, primarily focusing on metabolic pathways. Our simulations predicted that increasing the fraction of marine fatty acids in the diet, or increasing the dietary lipid/carbohydrate ratio resulted in (i) an increase in mitochondrial fatty acid beta oxidation, and (ii) changes in only a limited subset of mitochondrial reactions, which appeared to be independent of gene expression regulation. Moreover, transcript levels of mitochondrial proteins suggested unaltered fusion–fission dynamics by an increased lipid/carbohydrates ratio or by increased fractions of marine fatty acids. In conclusion, our enterocytic mitochondrial constraint-based model was shown to be a suitable platform to investigate effects of dietary interventions on mitochondrial adaptation and provided novel and deeper insights in mitochondrial metabolism and regulation.

## Introduction

The prevalence of obesity is rising globally the last decades, which can be mostly attributed to an increase in calorie intake, changes in the composition of diet, declining levels of physical activity, and changes in the gut microbiome (Ng et al., 2014). Cellular metabolism of the nutrients that are consumed depends largely on mitochondria (Nunnari and Suomalainen, 2012; Liesa and Shirihai, 2013), where, for instance, fatty acid beta oxidation and the tricarboxylic acid (TCA) cycle are located. Moreover, the mitochondrial electron transfer complexes conferring energy from substrate oxidation into ATP synthesis are crucial for cellular metabolic capacity (Nunnari and Suomalainen, 2012; Liesa and Shirihai, 2013). Increasing dietary fat intake causes a gradual increase in whole-body fat oxidation, a process called metabolic adaptation (Galgani et al., 2008; Hall and Guo, 2017). However, in the absence of a compensatory increase in energy expenditure, a positive energy balance is obtained that results in an increase in body fat mass (Leibel et al., 1995).

It has been reported that in mice fed a high-fat diet the uptake of fatty acids (FA) by the small intestine is increased (Petit et al., 2007), which is paralleled by induction of enterocytic genes and proteins involved in lipid metabolism (absorption, oxidation) and oxidative phosphorylation pathways, including carnitine palmitoyltransferase 1a (Cpt1a). In addition, types of fats [saturated vs. (poly)unsaturated] have a differential impact on small intestinal gene expression and fatty acid oxidation rates (Mori et al., 2007). Combined, these data show that intestinal lipid oxidative capacity was increased upon changes in amount and type of dietary fats. However, whether this is due to increased mitochondrial metabolism (i.e., activity) or increased mitochondrial density (i.e., capacity) cannot be disentangled by the currently available experimental data.

A constraint-based metabolic model provides a framework to analyze specific dietary conditions and their impact on, e.g., mitochondrial physiology. The constraint-based approach is founded on the fact that cellular networks are constrained to operate within boundaries set by physio-chemical constraints, such as mass conservation, directional flow, and enzymatic capacity. Constraint-based modeling procedures have been successful in predicting metabolic phenotypes for various organisms, including microbiota, humans, and mice (AbuOun et al., 2009; Heinken et al., 2013; Thiele et al., 2013; Fondi and Lio, 2015). In multicellular organisms, constraint-based models have been constructed for various cell types such as enterocytes (Sinha et al., 2017), hepatocytes (Bordbar et al., 2011a), cardiomyocytes (Thiele et al., 2005), macrophages (Bordbar et al., 2012), and red blood cells (Bordbar et al., 2011b). Models have also been developed to specifically investigate diet-dependent changes in, for instance, cardiac mitochondria (Thiele et al., 2005) or contractile and metabolic functions in cardiomyocyte mitochondria (Cortassa et al., 2006, 2009). A dynamic model of mitochondria has also been developed to address the impact of aging and metabolism on mitochondrial physiology (Kowald and Klipp, 2014). Additionally, dynamic models of selected pathways have been developed to investigate the impact of genetic disorders on mitochondrial FA oxidation (van Eunen et al., 2013, 2016). These models, however, were not able to address the overall physiology of the cells either due to lack of parameters, or inclusion of parameters that are difficult to measure. These two modeling approaches, dynamic models based on differential equations and constraint based models based on mass balances, have complementary strengths (Machado et al., 2012; Stalidzans et al., 2018). Dynamic models are able to account for transient changes in the metabolic state and are able to account for changes in concentrations of metabolites. In these models enzyme abundance and regulatory events are explicitly included. These models, however, require a large number of parameters that are often difficult to measure and as a result dynamic modeling cannot be applied to systems at the genome scale, which typically include hundreds of reactions. On the other hand, constraint-based approaches require a minimal number of parameters (stoichiometric coefficient and maximum possible fluxes) and can be extended to thousands of reactions. As a limitation, constraint based-models only describe average reaction rates attainable by cells grown in steady or slowly varying environmental conditions, so that only steady states can be described. Moreover, very limited regulatory information can be included in these models so that non-linear effects cannot be described. The aim of the present study was to study mitochondrial physiology of small intestinal enterocytes under different dietary conditions, using data of nutritional studies performed in mice. Therefore, a murine enterocyte-specific constraint-based mitochondrial metabolic model was constructed by combining and extending a recently published model of murine small intestinal epithelial cells with a model of human cardiomyocytes mitochondria (Vo et al., 2004; Sinha et al., 2017). To study lipid handling capacity of mitochondria in response to changes in dietary lipids composition and/or amount, modeling results were complemented by gene expression analysis of mitochondrial proteins.

## Materials and Methods

### Construction of a Constraint-Based Murine Enterocytic Mitochondrial Metabolic Model

First, all mitochondrial, cytosolic, and extracellular reactions were selected from a human constraint-based cardiomyocyte metabolic model (Vo et al., 2004; Thiele et al., 2005). Next, these reactions were manually curated using the constraint-based mouse-specific enterocyte metabolic model *mmu_ENT717* as reaction database (Sinha et al., 2017). Reactions for luminal carbohydrate and lipid absorption into small intestinal cells were also added. Finally, the model was crosschecked for missing genes for particular mitochondrial reactions using information from the MitoCarta 2.0 database (Calvo et al., 2016). The *mmu_ENT717* model contains a detailed description of lipid metabolism. Thus, the metabolism, including mitochondrial oxidation, of a variety of individual fatty acids and their cytoplasmic mono- (MAG), di- (DAG), and triacylglycerol (TAG) moieties can be modeled (Sinha et al., 2017). This approach was also implemented in the current enterocytic mitochondrial model. Myristic acid (C14:0), palmitic acid (C16:0), stearic acid (C18:0), oleic acid (C18:1), linoleic acid (C18:2), and linolenic acid (C18:3)] determine the whole spectrum of different lipids in the palm and soy oil-based diets used in one of the nutritional studies (see below). Moreover, the polyunsaturated (PUFA) omega-3 marine fatty acids eicosapentaenoic acid (EPA; C20:5) and docosahexaenoic acid (DHA; C22:6) and their associated MAG, DAG, and TAG moieties were also added to the model, enabling the specific investigation and modeling of enterocytic mitochondrial marine fatty acid import, handling, and oxidation of the fish oil-based diets. Here, it is assumed that all fatty acids can be imported into mitochondria and thus used for mitochondrial beta oxidation, as is suggested by indirect evidence of cardiolipin-containing very long-chain fatty acids in mitochondria (Shi, 2010).

Mitochondria consist of four distinct components, which from the cytoplasmic outside to the inside, are the mitochondrial outer membrane, the mitochondrial inter membrane space, mitochondrial inner membrane, and the mitochondrial matrix (Nunnari and Suomalainen, 2012; Liesa and Shirihai, 2013). Because the mitochondrial outer membrane is highly permeable for metabolites, no distinction is required for modeling between the intermembrane space, mitochondrial outer membrane, and the cytosol. Although the model was focused on the mitochondrial matrix, several cytosolic reactions were incorporated to support mitochondrial function. These include reactions representing the cytosolic side of the malate-aspartate, glycerol-phosphate, and carnitine shuttles, as well as the cytosolic steps required for heme synthesis. We checked the model integrity using function in the COBRA toolbox, which confirmed that the mitochondrial model was producing ATP and there were no dead-end metabolites (Schellenberger et al., 2011).

### Dietary Interventions

Data from our two previously published nutritional mouse studies was used. The first study focused on increased fractions of marine fatty acids, provided as triglycerides by partly replacing the other dietary omega-3 fatty acid, alpha-linolenic acid (C18:3). Intestinal transcriptomics dataset associated to this study is available at Gene Expression Omnibus (GEO) under accession number GSE11936 (van Schothorst et al., 2009). The second study investigated the impact on the small intestine of four fully characterized diets that mainly differed in ratio of lipids over carbohydrates. The experimental data used in this study were retrieved after 4 weeks of exposure to the new diets. Array data for this study are available under accession number GSE26300 (de Wit et al., 2011).

**FIGURE 1:**
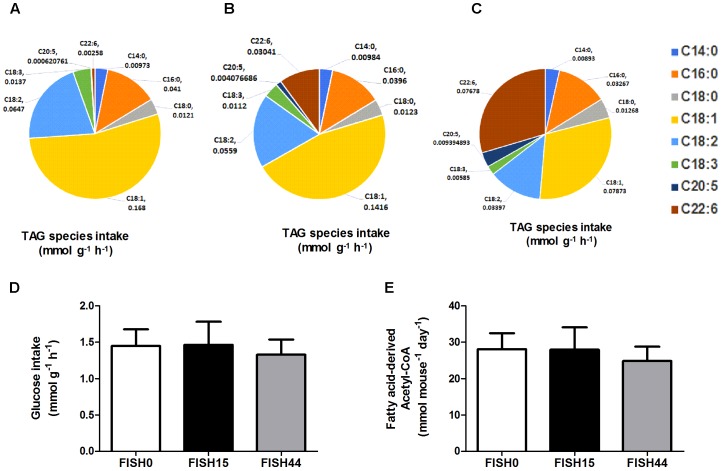
Fish diets nutrient intake. Maximal potential intake rates of the fatty acid species depicted as uniform triacylglycerides (TAG) present in **(A)** FISH0; **(B)** FISH15 diet; and **(C)** FISH44 diet. **(D)** Maximal potential intake rate of glucose in the FISH0, FISH15 and FISH44 diets; **(E)** Maximal possible fatty acid derived Acetyl–CoA in the FISH0, FISH15, and FISH44 diets. Rates in panels **A–D** are expressed as mmol per gram dry weight of intestinal tissue per hour (mmol g^-1^ h^-1^), in panel **E** as mmol per mouse per day. **(D,E)** Data are shown as mean ±*SD* (*n* = 10–12), and no significant differences were observed.

The marine fatty acid study (van Schothorst et al., 2009) is based on isocaloric high fat (60 en%) diets that differed only in composition of the omega-3 PUFA fraction. Mean dietary intake and fatty acid profiles have been reported before (Ruzickova et al., 2004; Flachs et al., 2005). The previously measured dietary fatty acid profile of the control diet (called cHF in Ruzickova et al., 2004), here called FISH0, is shown as intake in **Figure 1A**, taking food intake into account. As intervention diets, 15% or 44% of total fat component was replaced by fish oil (EPAX) rich in EPA and DHA; these diets were named cHF-F1 and cHF-F2, respectively (Ruzickova et al., 2004) and are called FISH15 and FISH44 in the current study. A description of the diets is shown in **Table 1**. The fatty acid profiles intake of these diets is shown in **Figures 1B,C**. Dietary maximal glucose intake based on daily food intake was not significantly different between these three diets (**Figure 1D**). Intestinal scrapings were used for whole-genome gene expression analysis (microarray) and for qPCR analysis of target genes, including Cpt1a, that encodes the mitochondrial fatty acid transporter that is rate limiting in mitochondrial fatty acid beta oxidation. Part of the small intestines was used for functional *ex vivo* fatty acid beta oxidation analysis (van Schothorst et al., 2009).

**Table 1:** Composition of diets differing in marine fatty acids or increased lipid/carbohydrate ratio.

	Fish fatty acids			Increased lipid/carbohydrate			
	FISH0	FISH15	FISH44	FAT10	FAT20	FAT30	FAT45
**Macronutrients (%, wt/wt)**							
Lipids	35.2	35.2	35.2	4.3	9.0	14.3	23.6
Carbohydrates	35.4	35.4	35.4	66.4	59.9	52.8	40.3
Proteins	20.5	20.5	20.5	19.0	20.0	21.2	23.3
Energy density (kJ/g)	22.8	22.8	22.8	16.1	17.0	18.0	19.8
**Lipid composition (%)**							
Rapeseed oil	95%	81%	53%				
Sunflower oil	5%	4%	3%				
EPAX 1050 TG	–	15%	44%				
Soybean oil				56%	28%	19%	12%
Palm oil				44%	72%	81%	88%
**FA composition of dietary lipids (g/100 g)**							
C14:0	2.4	2.4	2.4	0.9	1.5	1.6	1.8
C16:0	11.3	10.8	9.8	24.5	33.8	36.7	39.0
c18:0	3.7	3.7	4.2	4.0	4.0	4.0	4.0
C18:1	50.8	42.3	25.9	30.6	35.8	37.5	38.8
C18:2	19.4	16.6	11.1	35.2	22.6	18.6	15.4
C18:3	4.1	3.3	1.9	4.2	2.1	1.4	0.9
C20:5 (EPA)	0.2	1.3	3.3	–	–	–	–
C22:6 (DHA)	0.9	10.5	29.2	–	–	–	–
**Glucose derived from carbohydrates (g/100 g)**	31.3	31.3	31.3	65.3	57.7	49.2	34.4

*Dietary composition is given for macronutrients as weight %, with specifics for the lipid fraction (relative %) and subsequent fatty acid composition for the diets focused on marine fatty acids (FISH0, FISH15, and FISH44), and diets with increased lipids over carbohydrates ratio (FAT10, FAT20, FAT30, and FAT45), and total glucose availability derived from the carbohydrates sources. Details of these diets are previously reported by (Ruzickova et al., 2004; Flachs et al., 2005) for the marine fatty acid diets and by (de Wit et al., 2011) for the increased lipid/carbohydrate ratio diets*.

For the second intervention study, composition of the four diets and daily feed intake calculated from weekly intake levels have been reported previously (de Wit et al., 2011). Briefly, palm oil content was increased (from 19 to 207 g/kg) by exchange with starch (405 to 85 g/kg), while soy oil content (∼25 g/kg) and sucrose levels (∼170 g/kg) remained constant, giving rise to FAT10, FAT20, FAT30, and FAT45 diets, with the fat energy% denoted by the number. The ratio of palm versus soy oil varied between the diets because an increase in palm oil content was exchanged with starch, while soy oil content remained constant. As a result, the fatty acid profiles of the diets differed. These diets were free of cholesterol. All diets contained the same 20 energy% of casein as protein. The FAT10 diet was used as reference diet. Information on the conversion of dietary composition to dietary constrains used to simulate the model have been described previously (Sinha et al., 2017). Detailed information of the diets is provided in Supplementary Data S1.

### Modeling Metabolic Constraints Under Different Dietary Lipids Conditions

To calculate uptake rates for EPA, DHA, and glucose, the same approach was used as reported previously (Sinha et al., 2017). Uptake rates were then converted into mmol g^-1^ h^-1^ to constrain nine exchange reactions for the marine fatty acid containing diets (See Supplementary Data S1). Values from our previously published study (Sinha et al., 2017) were used to constrain the nine exchange reactions for the diets that differed in lipid/carbohydrates ratios, of which seven overlap. These exchange reactions are indicated in Supplementary Data S1. Under all dietary conditions, maximal ATP production was chosen as the metabolic objective for the simulations.

### Expression of Marker Genes for Mitochondrial Regulations

Transcriptome data from scraped small intestinal tissue from both studies (GSE11936 and GSE26300, respectively) was used to analyze differential expression of marker genes for mitochondrial dynamics by the dietary interventions. Normalized probe set expression estimates were computed by the GC-robust multiarray analysis (GC-RMA) algorithm as implemented in the Bioconductor package *affyPLM* (Wu et al., 2004; Bolstad et al., 2005). Probe sets were redefined based on annotations provided by the Entrez Gene database (custom CDF v21) (Dai et al., 2005). Differential expression was analyzed using the Bioconductor package *limma* (Ritchie et al., 2015). In each case, data corresponding to the interventions was compared with their respective controls.

### Sampling the Steady State

To characterize network capabilities under different dietary conditions the constraint-based murine small intestinal mitochondrial model was analyzed using a sampling approach. Sampling the steady-state flux space was performed using the random walk algorithm ACHR (artificial centering hit-and-run) (Kaufman and Smith, 1998). Sampling the solution space allows to investigate the flux distributions that satisfy the steady state condition. The ACHR algorithm method chooses an initial point within the solution space. It then calculates warm-up points from the initial point using several iterations of a basic hit-and-run algorithm (Schellenberger and Palsson, 2009). These warm-up points are stored as columns of a matrix *W*, and an approximate center, ***s***, is calculated. The direction for the next iteration from a sample point, ***x***_*m*_, is chosen by randomly taking one-point ***y*** out of the matrix *W* and applying the direction vector of ***y*** and ***s*** 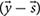 to ***x***_*m*_. At each iteration, the newly calculated point, ***x***_*m*+1_, is substituted randomly into *W* in the place of a previously calculated point (Schellenberger and Palsson, 2009). After each iteration, approximate values of center are recalculated.

Here, in each sampling procedure, 2000 randomly distributed points were computed with 500 iterations between each point. All sampling calculations were done in *MATLAB* *version R2015b* (The Mathworks Inc., Natick, MA, United States) using the COBRA toolbox and Gurobi solver version 6 (Bordbar et al., 2011a; Schellenberger et al., 2011).

### Statistical Analysis

All analyses were performed using *MATLAB (version R2015b)*, unless noted otherwise. Glucose intake and fatty acid-derived AcetylCoA intake were analyzed between groups using one-way ANOVA. For each reaction simulation, statistical analyses between groups of flux values compatible with the steady state were compared with one-way ANOVA, followed by the Tukey–Kramer *post hoc* test. Pearson correlation analysis was performed of quantitative RT-PCR and functional data of *Cpt1a* with functional FA beta oxidation rates (van Schothorst et al., 2009), both expressed as mean ± standard error of the mean (*SEM*, *n* = 9) after normalizing to the average of the FISH0 group.

## Results

To investigate mitochondrial adaptability and flexibility in small intestinal enterocytes, a constraint-based model of mitochondrial metabolism in murine enterocytes was constructed. The model was used to simulate mitochondrial behavior under different dietary conditions, representing various levels and composition of nutrients absorbed by the enterocytes. Two different nutritional intervention studies were used in the framework of the model. The first intervention represents an isocaloric exchange of mainly omega-3 alpha-linoleic acid by marine FA, containing eicosapentaenoic acid (EPA) and docosahexaenoic acid (DHA), in two different doses. The second nutritional intervention used four diets with increasing lipids to carbohydrate ratios.

**FIGURE 2:**
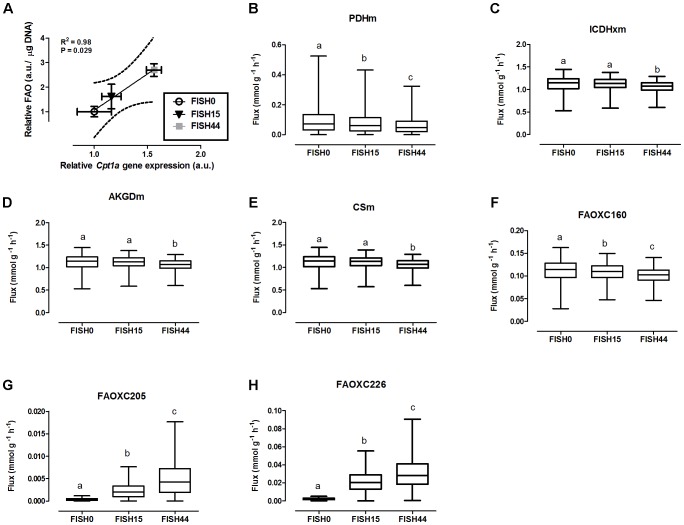
Simulated flux for selected mitochondrial reactions for the Fish diets. **(A)** Relative gene expression levels of *Cpt1a* (carnitine palmitoyltransferase 1a) versus relative functional fatty acid beta-oxidation level with the solid line representing the Pearson correlation line, and the dotted lines representing the 95% confidence interval. **(B–H)** The ACHR algorithm was used to sample the steady-state flux space for each diet, and results are presented in boxplots. Boxplot for simulated fluxes through **(B)** pyruvate dehydrogenase PDHm, **(C)** isocitrate dehydrogenase ICDHxm, **(D)** 2-oxoglutarate dehydrogenase AKGDm, and **(E)** citrate synthase CSm. Boxplots for simulated fluxes through **(F)** beta oxidation of palmitic acid FAOXC160, **(G)** beta oxidation of eicosapentaenoic acid FAOXC205, and **(H)** beta oxidation of docosahexaenoic acid FAOXC226. Boxplots represent median, upper, and lower quartile values of flux through the corresponding reactions for each diet, and data is expressed as mmol per gram dry weight of intestinal tissue per hour (mmol g^-1^ h^-1^). Different letters (a, b, c) indicate significant differences between the diets (*P* < 0.05) according to one-way ANOVA followed by Tukey–Kramer *post hoc* testing. Mean values with the same letter thus indicate no significant difference.

Data from the first study was included because it focused on dietary marine fatty acids, since those were previously shown to functionally induce intestinal fatty acid beta-oxidation (van Schothorst et al., 2009). Here, a control diet was based on rapeseed and sunflower oil as fat component, for which the dietary fatty acid profile intake is shown in **Figure 1A**. The content and composition of proteins and carbohydrates, including glucose, remained the same in all three diets; although small differences appeared in average daily food intake, this was not significantly different. As a result, also average glucose intake was similar (**Figure 1D**). Both transcript levels of *Cpt1a* and *ex-vivo* functional small intestinal fatty acid beta-oxidation were shown to be dose-dependently increased by the FISH15 and FISH44 diets (van Schothorst et al., 2009) and they are shown here to be significantly (*p* < 0.05) correlated (**Figure 2A**). This supports the notion that gene expression data can, at least for this process be used as proxy for functionality.

In the second study, diets with increased lipid to carbohydrates ratios were used. The amount of energy derived from lipids varied from FAT10 (lowest) to FAT45 (highest) energy%, and as a consequence, the carbohydrate content decreased from 69 to 35 energy%. The energy derived from protein was 20 energy% for all diets.

### Constraint-Based Model of Metabolism in Murine Enterocytic Mitochondria

As a starting point for the model construction, our previously published murine small intestinal enterocyte cellular metabolic model *mmu_ENT717* was used (Sinha et al., 2017). The model was subsequently restricted so that only reactions directly associated and required for mitochondrial function were kept. The resulting model was extensively curated focusing specifically on carbohydrate and lipid metabolism. Metabolic reactions for the two marine fatty acids EPA and DHA were also added. The resulting model is called *MT_mmuENT127* where “MT” stands for mitochondria and “*mmu_ENT*” stands for mouse enterocytes, and it comprises 127 metabolic genes, 178 unique metabolites, and 311 reactions. The model contains standardized reaction identifiers according to the BiGG Models database (King et al., 2016).

Although the model was focused on the mitochondrial matrix, it was necessary to incorporate some mitochondrial inner membrane and cytosolic reactions to support mitochondrial function and to describe cellular nutrient import and export. Thus, *MT_mmuENT127* comprises 3 compartments, being luminal “u”, cytosolic “c”, and mitochondrial “m”. The included reactions describe TCA cycle, mitochondrial fatty acid beta oxidation, phospholipid biosynthesis, and oxidative phosphorylation, among others. The reactions are distributed over the compartments: 24 luminal, 54 cytosolic, and 80 mitochondrial. There are 91 transport reactions introduced between the compartments and 62 exchange reactions were included into the model. Full details of the model can be found in Supplementary Data S2 and Supplementary Data S3. Enzymes anchored to the outer mitochondrial membrane with their activity located in the cytoplasm, for example acyl-transferases, were represented by their cytoplasmic compartment. Likewise, protons are pumped out from the mitochondrial matrix “m” by oxidative phosphorylation at complex I, III, and IV into the mitochondrial intermembrane space, thus denoted as cytoplasmic “c”.

**FIGURE 3:**
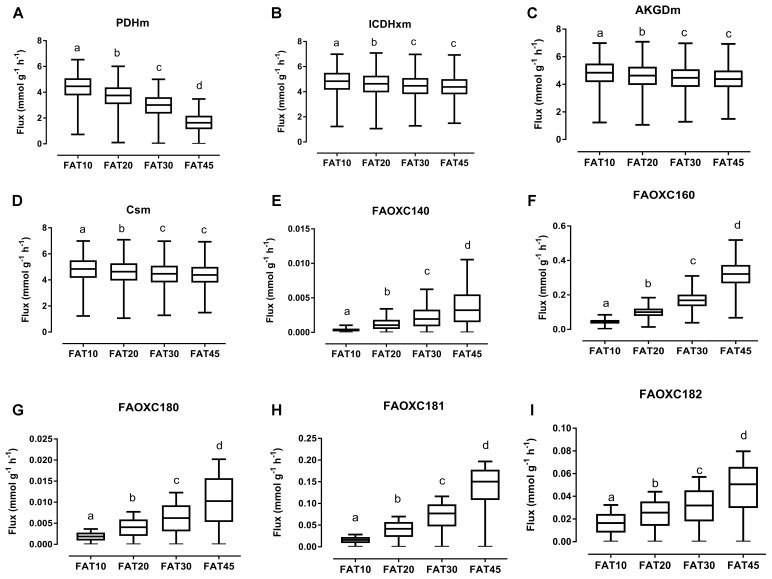
Simulated flux for selected mitochondrial reactions for the diets differing in lipid/ carbohydrates ratio. The ACHR algorithm was used to sample the steady-state flux space for each diet, and results are presented in boxplots. Boxplots for **s**imulated fluxes through **(A)** PDHm, **(B)** ICDHxm, **(C)** AKGDm, **(D)** CSm, **(E)** beta oxidation of myristic acid FAOXC140, **(F)** beta oxidation of palmitate FAOXC160, **(G)** beta oxidation of octadecanoic acid FAOXC180, **(H)** beta oxidation of oleic acid FAOXC181, and **(I)** beta oxidation of linoleic acid FAOXC182. Box plots represent median, upper, and lower quartile values of flux through the corresponding reactions for each diet, and data is expressed as mmol per gram dry weight of intestinal tissue per hour (mmol g^-1^ h^-1^). Different letters (a, b, c, d) indicate significant differences between the diets (*P* < 0.05) according to one-way ANOVA followed by Tukey–Kramer *post hoc* testing. Mean values with the same letter thus indicate no significant difference.

The consistency of the model was tested by assessing the integrity of the network and no gaps were found in the model. To evaluate the predictive potential of the *MT_mmuENT127* model, the capability of the model to produce ATP in mitochondria was tested as metabolic objective using both nutritional intervention studies. The model was seen to be able to carry flux through the reactions associated with carbohydrate metabolism Pyruvate dehydrogenase (PDHm), TCA metabolism shown by Isocitrate dehydrogenase (ICDHxm), Citrate synthase (CSm), and 2-oxoglutarate dehydrogenase (AKGDm), and fatty acid beta oxidation of long chain fatty acids (i.e., FAOXC160, FAOXC205, and FAOXC226) for the marine fatty acids diets as shown in **Figures 2B–H**. The model was also seen to carry flux under constraints associated to the four dietary interventions with increased ratios of lipids/carbohydrates for the reactions by PDHm, ICDHxm, and CSm for carbohydrate and TCA metabolism, and mitochondrial fatty acid beta oxidation reactions FAOXC140, FAOXC160, FAOXC180, FAOXC181, and FAOXC182 as shown in **Figures 3A–I**.

### Prediction of Mitochondrial Metabolic Adaptation Upon Different Dietary Lipids Interventions

To investigate mitochondrial adaptation by changes in enterocytic dietary nutrient availability, ACHR sampling of the steady-state flux space was used with maximal ATP synthesis as an objective function. By increasing the marine fatty acids content in the diet, a decreased flux through PDHm was observed (**Figure 2B**), together with a decreased flux through the TCA cycle as shown by the reduced flux through the two TCA cycle reactions that release carbon dioxide, i.e, (ICDHxm; **Figure 2C**) and (AKGDm; **Figure 2D**), and the rate limiting reaction catalyzed by CSm (**Figure 2E**). Of note, only the composition of the fatty acid species in the diets was changed from FISH0 to FISH15 to FISH44, resulting in increased EPA and DHA levels, but total amount of carbohydrates, fats, and protein were kept identical. Analysis of diet composition together with daily dietary intake, indeed, showed that total amount of acetyl-CoA solely derived from fatty acid beta oxidation appeared similar between FISH0, FISH15, and FISH44 diets (**Figure 1E**). Combined with a significant decreased availability of carbohydrate-derived acetyl-CoA (PDHm), it follows that also TCA cycle flux is decreased from FISH0 and FISH15 to FISH44 diet. Simultaneously, the model indicated that the beta-oxidation fluxes of the specific fatty acid species EPA (FAOXC205; **Figure 2G**) and DHA (FAOXC226; **Figure 2H**) increased as expected, while dietary palmitate oxidation (FAOXC160, **Figure 2F**) decreased. This latter observation should be viewed as solely the oxidation of dietary palmitate, not as palmitate being an intermediate of longer fatty acids beta oxidation.

As expected, diets with increasing lipid versus carbohydrate content significantly decreased the flux through mitochondrial PDHm, as shown in **Figure 3A**. Significant decreases in TCA flux through ICDHxm, AKGDm, and CSm were also found for the FAT20, FAT30, and FAT45 diets versus FAT10 control diet (**Figures 3B–D**, respectively). However, simultaneously the fluxes through each specific beta-oxidation reaction significantly increased: beta oxidation of FAOXC140, **Figure 3E**; FAOXC160, **Figure 3F**; FAOXC180, **Figure 3G**; FAOXC181, **Figure 3H**; and FAOXC182, **Figure 3I**.

### Changes in Gene Expression Profiles of Mitochondrial Metabolic Genes Present in Metabolic Model *MT_mmu-ENT127*

**FIGURE 4:**
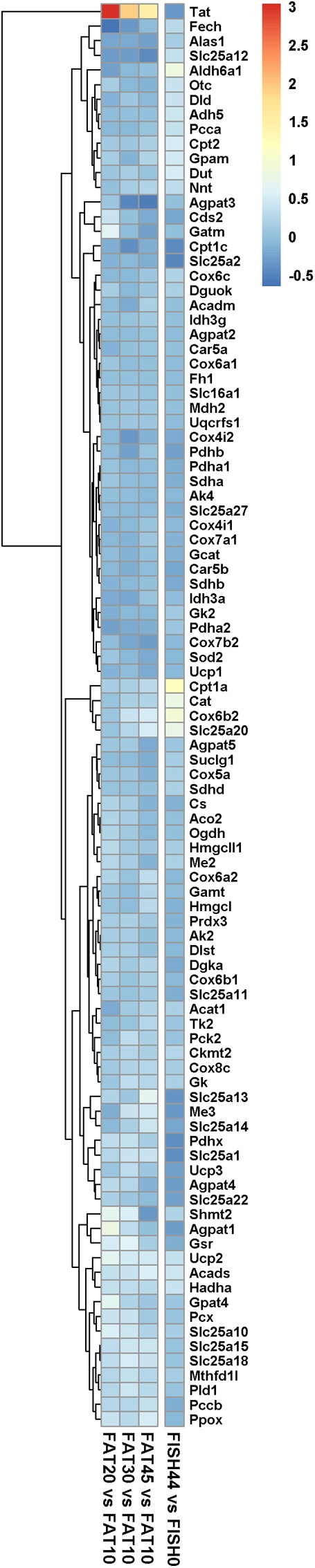
Expression of marker genes for mitochondrial activity and dynamics. Heat map representing the average log_2_ fold change of marker genes for mitochondrial activity and dynamics in intestines upon intervention with diets differing in lipid/ carbohydrates ratio, and FISH44 diet. Each diet was compared to its respective control, i.e., the FAT10 or FISH0. The color scale represents the log_2_ fold change of each gene; the more intense the color the more pronounced its regulation. Red means induced, whereas blue means suppressed in the experimental group compared to its control. Clustering is based on the data obtained by analysis of the increased lipid/carbohydrate ratio, and FISH44 versus FISH0 data is sorted to fit this clustering.

In both nutritional intervention studies, intestinal gene expression profiles were analyzed, focusing specifically on genes encoding mitochondrial located proteins. Remarkably, only a few mitochondrial genes showed changes in their expression levels for the FISH15 versus the control FISH0 group, whereas for the increased lipid/carbohydrate ratio diet groups, when comparing FAT20, FAT30, and FAT45 versus control FAT10 group (**Figure 4**) no significant changes were observed. This cumulatively suggests that the capacity of enterocytic mitochondria to handle a large range of fatty acid levels and/or composition is not regulated at the gene expression level. Focusing in detail on those genes that do show gene expression regulation, *Cat,* *Cox6b2,* *Slc25a20*, and *Cpt1a,* the latter two are both part of the carnitine shuttle system to import fatty acids into mitochondria, which were significantly increased by increased fish fatty acid levels supporting increased fatty acid beta oxidation (**Figures 2A**, **4**); expression levels of these genes were unaffected between FAT45 and FAT10 fat diets (**Figure 4**). As mitochondrial fusion-fission dynamics might underlie the adaptation of mitochondria to different fatty acids and/or their levels, we next focused on expression levels of genes specifically involved in these processes. However, all genes coding for proteins from the mitochondrial fusion-fission machinery, including Drp and Mfn, were not significantly regulated as shown in Supplementary Data S4. For comparison, also the log_2_ fold changes of FISH44 over control FISH0 are shown, which indicated extreme small changes for these transcripts, if at all present.

## Discussion

In this study we present a comprehensive and manually curated metabolic model of the mitochondria present in mice enterocytes, called *MT_mmu_ENT127*. This model is ideally suited to investigate adaptation and regulation of enterocytic mitochondria exposed to different types and load of lipids. Model simulations suggest different metabolic responses to diet changes associated to inclusion of fish fatty acids or by an increased lipid/carbohydrate ratio. Specifically, changes in PDHm or FA beta-oxidation fluxes differ between both types of exposures. This model can also be used to simulate the steady state reached after exposure to the modified diets.

The *MT_mmu_ENT127* model has an extensive representation of fatty acid metabolic reactions in enterocytic mitochondria. The *MT_mmuENT127* model was used to simulate for maximal ATP production (Stanley et al., 2005). Under increased lipid/carbohydrate ratio in the diets, results (as shown in **Figure 2**) indicate that with increased lipid intake there is a significant increase in FA beta oxidation flux of C14:0, C16:0, C18:0, C18:1, and C18:2 reactions, together with a decrease through PDHm. Analysis of different Fish diets showed that the model indicated less pronounced but similar patterns of a decreased flux through PDHm and an increase in fatty acid beta oxidation of the fish fatty acids EPA (C20:5) and DHA (C22:6). For the metabolic flux analyses as plotted in **Figures 2**, **3**, the model was set to simulate conditions of maximal ATP production.

Metabolic fluxes may be regulated by changes of gene (or protein) expression that adjust enzyme capacities (V_max_) and/or “metabolically” by interactions of enzymes with substrates (Fondi and Lio, 2015). Analysis of transcriptome data supports the overarching view that enterocytic mitochondria have enough intrinsic capacity to deal with the higher flux and the enzymes involved do not need to be regulated under increased lipid/carbohydrate ratio. Mitochondrial fusion and fission dynamics has been associated with changes in mitochondrial function and increased mitochondrial fusion leads to increased capacity and more efficient metabolic conversions (Putti et al., 2015). Nevertheless, all genes involved in mitochondrial fusion–fission dynamics were not significantly regulated at mRNA level as shown in Supplementary Data S4 [Gene expression]. As a marker for mitochondrial density, levels of several oxidative complexes are commonly used. Here, those were all not regulated (data not shown), supporting an absence of enterocyte mitochondrial density in its adaptation to different levels or composition of lipids and increased fatty acid beta oxidation.

The developed model describes cellular states in steady or slowly varying environmental conditions, thus the reported simulations are independent of each other and do not account for transient adaptations. These adaptations could differ when diets with a gradual increase of carbohydrate vs. lipid content are compared with diets with increased lipid over carbohydrate content. The increase in intestinal fatty acid beta oxidation flux levels and decrease in PDHm flux levels reflect higher fatty acid intake and lower glucose availability in the case of increased fat/carbohydrate ratio diets. At the cellular level, inhibition of the glycolytic pathway increases along the progression of the pathway, i.e. maximum inhibition at PDHm level and minimal at glucose uptake level, which fits with the modeling results (**Figure 2B**). PDHm is regulated by multiple mitochondrial effectors representing the energy state of the cell such as acetyl-CoA/CoA, ATP/ADP and NADH/NAD^+^ ratios. Other known regulators are Ca^2+^ and pyruvate (Cortassa et al., 2017), as well as protein modifications like (de-)phosphorylation and (de-)acetylation of PDHm. Inactivation by phosphorylation is mediated via pyruvate dehydrogenase kinases (PDK1-4). Activation of PDHm is performed by, e.g., Sirtuin 3, a NAD^+^ dependent mitochondrial deacetylase, thereby linking glycolysis with mitochondrial respiration (Ozden et al., 2014). Sirtuin 4, another member of the sirtuin family, was shown to diminish PDH activity via enzymatic hydrolysis of the lipoamide cofactors, lipoamidase activity, from the E2 component dihydrolipoyllysine acetyltransferase (DLAT) of PDH in cells and *in vivo*, in mouse liver (Mathias et al., 2014). Selection of substrates operates at the mitochondrial level when fatty acids and glucose are present in different ratios (Cortassa et al., 2017). Utilization of glucose and fatty acids are highly dependent on PDH activity and mitochondrial metabolism for substrate selection. In certain physiological conditions like type 2 diabetes, differential selection of substrate fuel shows greater rate of fatty acid oxidation and insulin resistance. Under this condition PDH is overexpressed in several tissues particularly in heart and skeletal muscle which reduces the consumption of glucose. On the contrary there is a shift in utilization of glucose as a substrate in case of aging heart for sustaining energy supply (Hansford, 1983; Lesnefsky et al., 2016). In case of surplus cellular energy, i.e., high acetyl-CoA/CoA and ATP/ADP ratios, acetyl-CoA will be redirected outside mitochondria *via* citrate, which in its turn inhibits phosphofructokinase, the rate-limiting step in glycolysis. Moreover, acetyl-CoA is used for FA synthesis via malonyl-CoA, again channeling surplus energy into fatty acid, e.g., storage, and simultaneously inhibiting fatty acid import into mitochondria as malonyl-CoA functionally inhibits CPT1a (Fraser et al., 1997; Shi and Tu, 2015). It should be kept in mind that enterocytes only use a (minor) part of the nutrients absorbed for their own cellular metabolism including oxidation for ATP production, while the majority of nutrients is secreted into circulation for the remainder of the body. Estimates for oxygen consumption by gastro-intestinal tract as percentage of whole body range from 11 to 14% for humans and rats, respectively, while their tissue mass versus body mass ranges from 2 to 5%, respectively (Rolfe and Brown, 1997). Especially in the post-absorptive phase, enterocytes use nutrients from circulation for ATP production, so the contribution of luminal absorbed nutrients for direct ATP production is difficult, if not impossible, to estimate.

**FIGURE 5:**
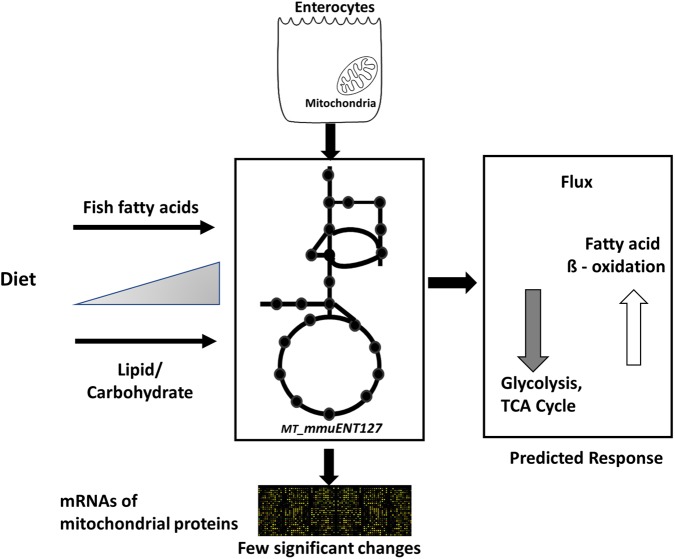
Overview of computational model and its response to different diets. Schematic representation of enterocyte-specific mitochondrial constraint-based metabolic model. The model was simulated with data derived from nutritional studies in mice using increased levels of type of lipids, i.e., fish fatty acids, and increased load of lipids by increased dietary lipid/carbohydrate ratios. The main physiological responses predicted by the model were an increase in fatty acid beta oxidation and a decrease in glycolysis and TCA cycle. Transcript levels of mitochondrial proteins indicated only a few significant changes in response to the diets.

Nonetheless, the results obtained by metabolic flux analysis are reflected in the gene expression analysis. For instance, increased fish fatty acids changed only a very few genes, such as *Cpt1a*, Catalase (*Cat*), solute carrier family 25 (carnitine/acylcarnitine translocase) member 20 *Slc25a20*, and cytochrome C oxidase subunit 6B2 (*Cox6b2)* (**Figure 4**), of which *Cpt1a* significantly positively correlated with functional intestinal beta oxidation (**Figure 1E**). This was reflected in the constraint model as well where only few genes associated to reactions changed at the expression level (**Figure 4**). This may be because the effective increases in protein levels are not reflected in the transcriptome as stated earlier. These findings are reflected in the *in silico* metabolic flux analysis as the decrease of PDHm flux and increase in beta oxidation flux levels were much less pronounced by inclusion of fish fatty acids than by an increased lipid/carbohydrate ratio. This difference is likely explained by the identical lipid load, although with different levels of fish fatty acids versus the more pronounced increased load of lipids by the diets with increased lipids/carbohydrate ratios. Unfortunately, no functional data of intestinal beta oxidation by increased lipid levels are available to compare effects at the functional level of specific fatty acids, here marine fatty acids, versus increases in dietary lipids. As an overall summary, the physiological conditions predicted by the model and its related responses is shown in **Figure 5**.

Our model does not predict any changes in mitochondrial physiology under the influence of gut microbiota with respect to mitochondrial redox metabolism and reactive oxygen species generation. Mitochondria do influence the activities of intestinal cells such as immune cells and epithelial cells (Clark and Mach, 2017).Under the influence of gut microbiota the functioning of mitochondria may become more evident.

In this study we present a comprehensive and manually curated metabolic model of the mitochondria present in mice enterocytes, called *MT_mmu_ENT127*. This model is ideally suited to investigate adaptation and regulation of enterocytic mitochondria exposed to different types and load of lipids. Model simulations suggest different metabolic responses to diet changes associated to inclusion of fish fatty acids or by an increased lipid/carbohydrate ratio. Specifically, changes in PDHm or beta-oxidation fluxes differ between both types of exposures, all without gross effects on transcript levels.

## Author Contributions

NS, MS-D, GH and EvS conceived the work and drafted the manuscript. NS, MS-D and VMdS generated the model and performed the computational analysis. JK, GH, and EvS provided data, performed the data processing and provided biological input for model creation and result interpretation. VMdS and JK provided intellectual input on the manuscript. All authors corrected and approved the manuscript.

## Conflict of Interest Statement

VMdS is affiliated to LifeGlimmer GmbH. The remaining authors declare that the research was conducted in the absence of any commercial or financial relationships that could be construed as a potential conflict of interest.

## References

[B1] AbuOun M. Suthers P. F. Jones G. I. Carter B. R. Saunders M. P. Maranas C. D. (2009). Genome scale reconstruction of a *Salmonella* metabolic model: comparison of similarity and differences with a commensal *Escherichia coli* strain. *J. Biol. Chem.* 284 29480–29488. 10.1074/jbc.M109.005868 19690172 PMC2785581

[B2] Bolstad B. M. Collin F. Brettschneider J. Simpson K. Cope L. Irizarry R. (2005). “Quality assessment of Affymetrix GeneChip data,” in *Bioinformatics and Computational Biology Solutions Using R and Bioconductor*, eds Gentleman R. Carey V. Huber W. Irizarry R. Dudoit S. (New York, NY: Springer), 33–47. 10.1007/0-387-29362-0_3

[B3] Bordbar A. Feist A. M. Usaite-Black R. Woodcock J. Palsson B. O. Famili I. (2011a). A multi-tissue type genome-scale metabolic network for analysis of whole-body systems physiology. *BMC Syst. Biol.* 5:180. 10.1186/1752-0509-5-180 22041191 PMC3219569

[B4] Bordbar A. Jamshidi N. Palsson B. O. (2011b). iAB-RBC-283: a proteomically derived knowledge-base of erythrocyte metabolism that can be used to simulate its physiological and patho-physiological states. *BMC Syst. Biol.* 5:110. 10.1186/1752-0509-5-110 21749716 PMC3158119

[B5] Bordbar A. Mo M. L. Nakayasu E. S. Schrimpe-Rutledge A. C. Kim Y. M. Metz T. O. (2012). Model-driven multi-omic data analysis elucidates metabolic immunomodulators of macrophage activation. *Mol. Syst. Biol.* 8:558. 10.1038/msb.2012.21 22735334 PMC3397418

[B6] Calvo S. E. Clauser K. R. Mootha V. K. (2016). MitoCarta2.0: an updated inventory of mammalian mitochondrial proteins. *Nucleic Acids Res.* 44 D1251–D1257. 10.1093/nar/gkv1003 26450961 PMC4702768

[B7] Clark A. Mach N. (2017). The Crosstalk between the gut microbiota and mitochondria during exercise. *Front. Physiol.* 8:319 10.3389/fphys.2017.00319 PMC5437217 28579962

[B8] Cortassa S. Aon M. A. O’rourke B. Jacques R. Tseng H. J. Marban E. (2006). A computational model integrating electrophysiology, contraction, and mitochondrial bioenergetics in the ventricular myocyte. *Biophys. J.* 91 1564–1589. 10.1529/biophysj.105.076174 16679365 PMC1518641

[B9] Cortassa S. O’rourke B. Winslow R. L. Aon M. A. (2009). Control and regulation of mitochondrial energetics in an integrated model of cardiomyocyte function. *Biophys. J.* 96 2466–2478. 10.1016/j.bpj.2008.12.3893 19289071 PMC2989151

[B10] Cortassa S. Sollott S. J. Aon M. A. (2017). “Substrate selection and its impact on mitochondrial respiration and redox,” in *Molecular Basis for Mitochondrial Signaling*, ed. Rostovtseva T. K. (Cham: Springer International Publishing), 349–375. 10.1007/978-3-319-55539-3_13

[B11] Dai M. Wang P. Boyd A. D. Kostov G. Athey B. Jones E. G. (2005). Evolving gene/transcript definitions significantly alter the interpretation of GeneChip data. *Nucleic Acids Res.* 33:e175. 10.1093/nar/gni179 16284200 PMC1283542

[B12] de Wit N. J. Boekschoten M. V. Bachmair E. M. Hooiveld G. J. De Groot P. J. Rubio-Aliaga I. (2011). Dose-dependent effects of dietary fat on development of obesity in relation to intestinal differential gene expression in C57BL/6J mice. *PLoS One* 6:e19145. 10.1371/journal.pone.0019145 21547079 PMC3081848

[B13] Flachs P. Horakova O. Brauner P. Rossmeisl M. Pecina P. Franssen-Van Hal N. (2005). Polyunsaturated fatty acids of marine origin upregulate mitochondrial biogenesis and induce beta-oxidation in white fat. *Diabetologia* 48 2365–2375. 10.1007/s00125-005-1944-7 16205884

[B14] Fondi M. Lio P. (2015). Genome-scale metabolic network reconstruction. *Methods Mol. Biol.* 1231 233–256. 10.1007/978-1-4939-1720-4_15 25343869

[B15] Fraser F. Corstorphine C. G. Zammit V. A. (1997). Topology of carnitine palmitoyltransferase I in the mitochondrial outer membrane. *Biochem. J.* 323(Pt 3), 711–718. 10.1042/bj3230711 PMC1218374 9169604

[B16] Galgani J. E. Moro C. Ravussin E. (2008). Metabolic flexibility and insulin resistance. *Am. J. Physiol. Endocrinol. Metab.* 295 E1009–E1017. 10.1152/ajpendo.90558.2008 18765680 PMC2584808

[B17] Hall K. D. Guo J. (2017). Obesity energetics: body weight regulation and the effects of diet composition. *Gastroenterology* 152:e1713. 10.1053/j.gastro.2017.01.052 28193517 PMC5568065

[B18] Hansford R. G. (1983). Bioenergetics in aging. *Biochim. Biophys. Acta* 726 41–80. 10.1016/0304-4173(83)90010-1 6338921

[B19] Heinken A. Sahoo S. Fleming R. M. Thiele I. (2013). Systems-level characterization of a host-microbe metabolic symbiosis in the mammalian gut. *Gut Microbes* 4 28–40. 10.4161/gmic.22370 23022739 PMC3555882

[B20] Kaufman D. E. Smith R. L. (1998). Direction choice for accelerated convergence in hit-and-run sampling. *Oper. Res.* 46 84–95. 10.1287/opre.46.1.84

[B21] King Z. A. Lu J. Drager A. Miller P. Federowicz S. Lerman J. A. (2016). BiGG Models: a platform for integrating, standardizing and sharing genome-scale models. *Nucleic Acids Res.* 44 D515–D522. 10.1093/nar/gkv1049 26476456 PMC4702785

[B22] Kowald A. Klipp E. (2014). Mathematical models of mitochondrial aging and dynamics. *Prog. Mol. Biol. Transl. Sci.* 127 63–92. 10.1016/B978-0-12-394625-6.00003-9 25149214

[B23] Leibel R. L. Rosenbaum M. Hirsch J. (1995). Changes in energy expenditure resulting from altered body weight. *N. Engl. J. Med.* 332 621–628. 10.1056/NEJM199503093321001 7632212

[B24] Lesnefsky E. J. Chen Q. Hoppel C. L. (2016). Mitochondrial metabolism in aging heart. *Circ. Res.* 118 1593–1611. 10.1161/CIRCRESAHA.116.307505 27174952 PMC5009371

[B25] Liesa M. Shirihai O. S. (2013). Mitochondrial dynamics in the regulation of nutrient utilization and energy expenditure. *Cell Metab.* 17 491–506. 10.1016/j.cmet.2013.03.002 23562075 PMC5967396

[B26] Machado D. Costa R. S. Ferreira E. C. Rocha I. Tidor B. (2012). Exploring the gap between dynamic and constraint-based models of metabolism. *Metab. Eng.* 14 112–119. 10.1016/j.ymben.2012.01.003 22306209 PMC3465724

[B27] Mathias R. A. Greco T. M. Oberstein A. Budayeva H. G. Chakrabarti R. Rowland E. A. (2014). Sirtuin 4 is a lipoamidase regulating pyruvate dehydrogenase complex activity. *Cell* 159 1615–1625. 10.1016/j.cell.2014.11.046 25525879 PMC4344121

[B28] Mori T. Kondo H. Hase T. Tokimitsu I. Murase T. (2007). Dietary fish oil upregulates intestinal lipid metabolism and reduces body weight gain in C57BL/6J mice. *J. Nutr.* 137 2629–2634. 10.1093/jn/137.12.2629 18029475

[B29] Ng M. Fleming T. Robinson M. Thomson B. Graetz N. Margono C. (2014). Global, regional, and national prevalence of overweight and obesity in children and adults during 1980-2013: a systematic analysis for the Global Burden of Disease Study 2013. *Lancet* 384 766–781. 10.1016/S0140-6736(14)60460-8 24880830 PMC4624264

[B30] Nunnari J. Suomalainen A. (2012). Mitochondria: in sickness and in health. *Cell* 148 1145–1159. 10.1016/j.cell.2012.02.035 22424226 PMC5381524

[B31] Ozden O. Park S. H. Wagner B. A. Song H. Y. Zhu Y. Vassilopoulos A. (2014). SIRT3 deacetylates and increases pyruvate dehydrogenase activity in cancer cells. *Free Radic. Biol. Med.* 76 163–172. 10.1016/j.freeradbiomed.2014.08.001 25152236 PMC4364304

[B32] Petit V. Arnould L. Martin P. Monnot M. C. Pineau T. Besnard P. (2007). Chronic high-fat diet affects intestinal fat absorption and postprandial triglyceride levels in the mouse. *J. Lipid Res.* 48 278–287. 10.1194/jlr.M600283-JLR200 17114807

[B33] Putti R. Sica R. Migliaccio V. Lionetti L. (2015). Diet impact on mitochondrial bioenergetics and dynamics. *Front. Physiol.* 6:109 10.3389/fphys.2015.00109 PMC4389347 25904870

[B34] Ritchie M. E. Phipson B. Wu D. Hu Y. Law C. W. Shi W. (2015). limma powers differential expression analyses for RNA-sequencing and microarray studies. *Nucleic Acids Res.* 43:e47. 10.1093/nar/gkv007 25605792 PMC4402510

[B35] Rolfe D. F. Brown G. C. (1997). Cellular energy utilization and molecular origin of standard metabolic rate in mammals. *Physiol. Rev.* 77 731–758. 10.1152/physrev.1997.77.3.731 9234964

[B36] Ruzickova J. Rossmeisl M. Prazak T. Flachs P. Sponarova J. Veck M. (2004). Omega-3 PUFA of marine origin limit diet-induced obesity in mice by reducing cellularity of adipose tissue. *Lipids* 39 1177–1185. 10.1007/s11745-004-1345-9 15736913

[B37] Schellenberger J. Palsson B. O. (2009). Use of randomized sampling for analysis of metabolic networks. *J. Biol. Chem.* 284 5457–5461. 10.1074/jbc.R800048200 18940807

[B38] Schellenberger J. Que R. Fleming R. M. Thiele I. Orth J. D. Feist A. M. (2011). Quantitative prediction of cellular metabolism with constraint-based models: the COBRA Toolbox v2.0. *Nat. Protoc.* 6 1290–1307. 10.1038/nprot.2011.308 21886097 PMC3319681

[B39] Shi L. Tu B. P. (2015). Acetyl-CoA and the regulation of metabolism: mechanisms and consequences. *Curr. Opin. Cell Biol.* 33 125–131. 10.1016/j.ceb.2015.02.003 25703630 PMC4380630

[B40] Shi Y. (2010). Emerging roles of cardiolipin remodeling in mitochondrial dysfunction associated with diabetes, obesity, and cardiovascular diseases. *J. Biomed. Res.* 24 6–15. 10.1016/S1674-8301(10)60003-6 23554606 PMC3596530

[B41] Sinha N. Suarez-Diez M. Van Schothorst E. M. Keijer J. Martins Dos Santos V. A. P. Hooiveld G. J. E. J. (2017). Predicting the murine enterocyte metabolic response to diets that differ in lipid and carbohydrate composition. *Sci. Rep.* 7:8784. 10.1038/s41598-017-07350-1 28821741 PMC5562867

[B42] Stalidzans E. Seiman A. Peebo K. Komasilovs V. Pentjuss A. (2018). Model-based metabolism design: constraints for kinetic and stoichiometric models. *Biochem. Soc. Trans.* 46 261–267. 10.1042/BST20170263 29472367 PMC5906704

[B43] Stanley W. C. Recchia F. A. Lopaschuk G. D. (2005). Myocardial substrate metabolism in the normal and failing heart. *Physiol. Rev.* 85 1093–1129. 10.1152/physrev.00006.2004 15987803

[B44] Thiele I. Price N. D. Vo T. D. Palsson B. O. (2005). Candidate metabolic network states in human mitochondria. Impact of diabetes, ischemia, and diet. *J. Biol. Chem.* 280 11683–11695. 10.1074/jbc.M409072200 15572364

[B45] Thiele I. Swainston N. Fleming R. M. Hoppe A. Sahoo S. Aurich M. K. (2013). A community-driven global reconstruction of human metabolism. *Nat. Biotechnol.* 31 419–425. 10.1038/nbt.2488 23455439 PMC3856361

[B46] van Eunen K. Simons S. M. Gerding A. Bleeker A. Den Besten G. Touw C. M. (2013). Biochemical competition makes fatty-acid beta-oxidation vulnerable to substrate overload. *PLoS Comput. Biol.* 9:e1003186. 10.1371/journal.pcbi.1003186 23966849 PMC3744394

[B47] van Eunen K. Volker-Touw C. M. Gerding A. Bleeker A. Wolters J. C. Van Rijt W. J. (2016). Living on the edge: substrate competition explains loss of robustness in mitochondrial fatty-acid oxidation disorders. *BMC Biol.* 14:107. 10.1186/s12915-016-0327-5 27927213 PMC5142382

[B48] van Schothorst E. M. Flachs P. Franssen-Van Hal N. L. Kuda O. Bunschoten A. Molthoff J. (2009). Induction of lipid oxidation by polyunsaturated fatty acids of marine origin in small intestine of mice fed a high-fat diet. *BMC Genomics* 10:110. 10.1186/1471-2164-10-110 19284886 PMC2662879

[B49] Vo T. D. Greenberg H. J. Palsson B. O. (2004). Reconstruction and functional characterization of the human mitochondrial metabolic network based on proteomic and biochemical data. *J. Biol. Chem.* 279 39532–39540. 10.1074/jbc.M403782200 15205464

[B50] Wu Z. Irizarry R. A. Gentleman R. Martinez-Murillo F. Spencer F. (2004). A model-based background adjustment for oligonucleotide expression arrays. *J. Am. Stat. Assoc.* 99 909–917. 10.1198/016214504000000683

